# Mapping Chromatin Occupancy of *Ppp1r1b-lncRNA* Genome-Wide Using Chromatin Isolation by RNA Purification (ChIRP)-seq

**DOI:** 10.3390/cells12242805

**Published:** 2023-12-08

**Authors:** John Hwang, Xuedong Kang, Charlotte Wolf, Marlin Touma

**Affiliations:** 1 Neonatal/Congenital Heart Laboratory, Cardiovascular Research Laboratories, David Geffen School of Medicine, University of California Los Angeles, Los Angeles, CA 90095, USA; johnhwang@mednet.ucla.edu (J.H.); xkang@mednet.ucla.edu (X.K.); cgwolf@g.ucla.edu (C.W.); 2 Department of Pediatrics, David Geffen School of Medicine, University of California Los Angeles, Los Angeles, CA 90095, USA; 3 Children’s Discovery and Innovation Institute, Department of Pediatrics, David Geffen School of Medicine, University of California Los Angeles, Los Angeles, CA 90095, USA; 4 Medical and Life Science, College of Life Science, University of California Los Angeles, Los Angeles, CA 90095, USA; 5 Molecular Biology Institute, College of Life Science, University of California Los Angeles, Los Angeles, CA 90095, USA; 6 Eli and Edythe Broad Stem Cell Research Center, David Geffen School of Medicine, University of California Los Angeles, Los Angeles, CA 90095, USA

**Keywords:** *Ppp1r1b-lncRNA*, long non-coding RNA, chromatin isolation by RNA purification, epigenetics, chromatin occupancy

## Abstract

Long non-coding RNA (lncRNA) mediated transcriptional regulation is increasingly recognized as an important gene regulatory mechanism during development and disease. LncRNAs are emerging as critical regulators of chromatin state; yet the nature and the extent of their interactions with chromatin remain to be fully revealed. We have previously identified *Ppp1r1b-lncRNA* as an essential epigenetic regulator of myogenic differentiation in cardiac and skeletal myocytes in mice and humans. We further demonstrated that *Ppp1r1b-lncRNA* function is mediated by the interaction with the chromatin-modifying complex polycomb repressive complex 2 (PRC2) at the promoter of myogenic differentiation transcription factors, *TBX5* and *MyoD1*. Herein, we employed unbiased chromatin isolation by RNA purification (ChIRP) and high throughput sequencing to map the repertoire of *Ppp1r1b-lncRNA* chromatin occupancy genome-wide in the mouse muscle myoblast cell line. We uncovered a total of 99732 true peaks corresponding to *Ppp1r1b-lncRNA* binding sites at high confidence (*p*-value < 1E-5) and enrichment score ≥ 10). The *Ppp1r1b-lncRNA*-binding sites averaged 558 bp in length and were distributed widely within the coding and non-coding regions of the genome. Approximately 46% of these true peaks were mapped to gene elements, of which 1180 were mapped to experimentally validated promoter sequences. Importantly, the promoter-mapped binding sites were enriched in myogenic transcription factors and heart development while exhibiting focal interactions with known motifs of proximal promoters and transcription initiation by RNA Pol-II, including TATA-box, transcription initiator motif, CCAAT-box, and GC-box, supporting *Ppp1r1b-lncRNA* role in transcription initiation of myogenic regulators. Remarkably, nearly 40% of *Ppp1r1b-lncRNA*-binding sites mapped to gene introns were enriched with the Homeobox family of transcription factors and exhibited TA-rich motif sequences, suggesting potential motif-specific *Ppp1r1b-lncRNA*-bound introns. Lastly, more than 136521 enhancer sequences were detected in *Ppp1r1b-lncRNA*-occupancy sites at high confidence. Among these enhancers, 3390 (12%) exhibited cell type/tissue-specific enrichment in fetal heart and muscles. Together, our findings provide further insights into the genome-wide *Ppp1r1b-lncRNA:* Chromatin interactome that may dictate its function in myogenic differentiation and potentially other cellular and biological processes.

## 1. Introduction

The majority of the mammalian genome is transcribed to produce RNA transcripts, most of which display no protein-coding potential [1]. Long noncoding RNA (lncRNA) transcripts define an expanding class of non-coding RNA species that are longer than 200 nucleotides and lack functional open reading frames. Like mRNAs, lncRNAs are primarily transcribed by RNA polymerase II (RNA Pol-II), 5′-capped, poly A-tailed, and post-transcriptionally modified mostly by splicing [2,3].

LncRNAs are pervasively transcribed across the genome and have emerged as important transcriptional regulators, affecting all layers of transcriptome regulation, including RNA transcription, splicing, and metabolism [2,3,4,5]. As our understanding of biochemical properties and functional diversity of lncRNA continues to evolve, it is widely accepted that lncRNAs can exert diverse functions that arise from their ability to form complex secondary structures with DNA-, RNA-, and protein-binding properties, leading to complex RNA-DNA, RNA-RNA, or RNA-protein interactions [5,6]. Moreover, a single lncRNA may contain several binding loops that are able to bind to nucleic acids via base pairing or to proteins by certain RNA binding motifs, thus allowing the coordination of signals between different types of macromolecules and chromatin-modifying complexes [5,6]. It has been evident that several lncRNAs, such as HOTAIR (HOX antisense intergenic RNA) and Bvht (Braveheart), can execute their regulatory functions by recruiting chromatin modification complexes and altering the state of chromatin accessibility, leading to transcriptional activation or repression [7,8]. By performing these diverse functions, lncRNAs can influence cellular biology, molecular processes, and tissue homeostasis at multiple levels, including transcriptome regulation, molecular networking, cellular differentiation, and developmental decisions [2,3,4,5,6,7,8].

During development, chromatin state is a key determinant of cellular differentiation, identity, and fate [9,10,11,12]. We have previously identified *Ppp1r1b-lncRNA* as an essential and functionally conserved epigenetic regulator of myogenic differentiation of cardiac and skeletal myocytes in both mice and humans [13]. Importantly, in response to *Ppp1r1b-lncRNA* loss, human induced pluripotent stem cells (hiPSCs)-derived cardiac progenitors and skeletal myoblast cell lines failed to produce early markers of myogenic differentiation program upon induction [13]. Cellular differentiation requires the activation of specific transcriptional programs that are governed by cell-specific master regulators and transcription factors [14,15]. We have demonstrated that *Ppp1r1b-lncRNA* interferes with polycomb repressive complex 2 (PRC2) binding at target promoters of the master transcription factors of myogenic differentiation, *TBX5* and *MyoD1*, leading to decreased enrichment of H3K27me3, a PRC2-catalyzed epigenetic marker of transcriptional repression. In turn, the resulting enhanced chromatin accessibility leads to positive regulation of *TBX5* and *MyoD1* and induction of myogenic differentiation programs in cardiac and skeletal myocytes. These findings support the key role of *Ppp1r1b-lncRNA* in modulating chromatin states in a gene-specific manner to promote myogenic differentiation.

Interestingly, while *Ppp1r1b-lncRNA* was initially thought to act locally on a neighboring protein-coding gene [3], our mechanistic studies, including chromatin isolation by RNA purification-polymerase chain reactions (ChIRP-PCR), revealed that *Ppp1r1b-lncRNA* executes its function by physically interacting with distantly located transcription factors (*TBX5* and *MyoD1*). In our work presented here, we uncover the full panel of *Ppp1r1b-lncRNA*-binding sites and explore how the specificity for *Ppp1r1b-lncRNA* interactions is achieved [13].

We employed a ChIRP strategy followed by single-read high throughput DNA sequencing and subsequent bioinformatics tools to map *Ppp1r1b-lncRNA* occupancy at the genome scale. By applying downstream peak calling pipeline and peak mapping to gene elements, we revealed genome-wide *Ppp1r1b-lncRNA*-bound chromatin and gained further insights into the specific motifs that may underlie *Ppp1r1b-lncRNA* function at proximal promoters or distant enhancers of its putative target genes, including those encoding myogenic differentiation factors, transcription regulation, and chromatin modifiers.

## 2. Materials and Methods

### 2.1. ChIRP Assay

#### 2.1.1. Probe Design for ChIRP

**Figure 1:**
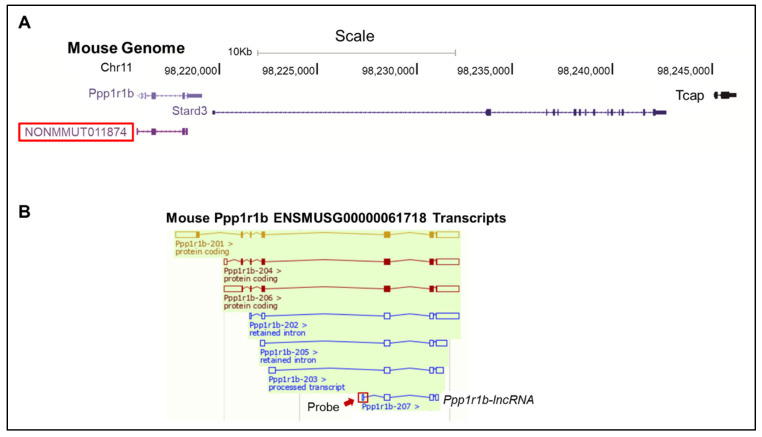
*Ppp1r1b-lncRNA* Genomic Position and Probe Design. (**A**) Schematic illustration of the relative position of lncRNA NONMMUT011874 *(Ppp1r1b-lncRNA)* in relation to *Ppp1r1b* gene in mouse genome. Adopted from Touma et al. 2016 [3]. (**B**) Mouse *Ppp1r1b* ENSMUSG00000061718 Transcripts. Red arrow and box highlight the unique exon that was targeted to design a *Ppp1r1b-lncRNA* anti-sense oligo probe for *Ppp1r1b-lncRNA*.

The Magna ChIRP RNA interactome kit (EMD Millipore Corp, Burlington, MA, USA) was used. Assays were performed per the manufacturer’s protocol. The capture probe is an antisense-oligo high-affinity probe targeted against a unique *Ppp1r1b-lncRNA* sequence (Figure 1) that does not overlap with other *Ppp1r1b* transcripts. It was designed using Stellaris Probe Designer version 1.0 (http://www.singlemoleculefish.com, accessed on 10 January 2018). The probe was compared with the mouse genome using the BLAT tool, https://www.ncbi.nlm.nih.gov/pmc/articles/PMC187518/ and no noticeable homology to non-*Ppp1r1b-lncRNA* targets were detected. An anti-sense oligo probe against lacZ RNA was provided by the ChIRP kit and used as a negative control for ChIRP-PCR experiments. Both probes were biotinylated at the 3′ end.

#### 2.1.2. Cell Culture

Mouse myoblasts, C2C12 cell line (ATCC), were cultured in DMEM (Invitrogen) supplemented with 10% Fetal Bovine Serum (FBS) and 1% Pen/Strep (Invitrogen).

#### 2.1.3. Cross-Linking, Sonication, and Hybridization

C2C12 cells were grown to log-phase in tissue culture plates and rinsed once with room temperature Phosphate buffer saline (Pbs). Cells were treated with glutaraldehyde for cross-linking, as described previously [13,16,17]. The resulting chromatin was fragmented by sonication. A sample consisting of 2% of the total input chromatin was then removed and served as the sequencing control. A biotinylated complementary anti-sense oligo probe was hybridized to *Ppp1r1b-lncRNA* and then isolated using magnetic streptavidin beads. No cross-hybridization with the LacZ probe was detected. The co-purified *Ppp1r1b-lncRNA* bound chromatin was then eluted for protein, RNA, and DNA. Using a combination of RNase A and RNase H, the DNA was gently eluted off of beads as described by the manufacturer’s instructions and processed into small fragments for library preparation.

### 2.2. ChIRP-Seq

#### 2.2.1. Library Preparation and High-Throughput Sequencing

The sequencing libraries were constructed from the ChIRP-captured and control “input” DNA fragments. Around 3 ng *Ppp1r1b-lncRNA*-ChIRP DNA and 3 ng control DNA “input” were used for library preparation as per manufacturers’ protocol. DNA fragments were subjected to DNA-end repair, 3′-ad overhanging, and adaptor ligation and then amplified using PCR. After size selection (between 100 and 500 bp), qualified *Ppp1r1b-ncRNA*-ChIRP and control DNA libraries were used for high throughput single-end (SE) sequencing on BGIseq at a read length of 50 bp, generating an average of 38 million raw sequencing reads per sample.

#### 2.2.2. Bioinformatic Analysis Workflow

1. Data filtering: Raw sequencing data were filtered using the software Short Oligonucleotide Analysis Package (SOAP) nuke 2.1.8 to remove adapter sequences, contamination, and low-quality reads. The following parameters were used for the SOAPnuke filter: I 5 q 0.5 -n 0.1 -Q 2 -c 40. Reads were considered “low-quality” if any of the following was true: (1) the ratio of N (unmappable reads) in whole read was >10%; (2) reads in which unknown bases exceeded 10%; or (3) the ratio of base whose quality was less than 20 was >10%.

2. Reads alignment: Clean reads that passed quality check measures were stored in FASTQ format and then aligned to the reference genome GRCm38/mm10 (Genome Reference Consortium Mouse Build 38 Organism: Mus musculus 10) using SOAP aligner SOAP2 (Version: 2.21t) [18]. No more than two mismatches were allowed in read alignment. Base coverage was normalized per million mappable reads. Reads from *Ppp1r1b-lncRNA*-ChIRP and control samples were aligned separately. The alignment results were then used for peak calling.

3. Peak calling and identifying true peaks: The uniquely mapped clean reads resulting from the alignment step were then used for peak calling. Candidate peaks for each sample were called using the software Model-Based Analysis for ChIP-Seq (MACS) v1.4.2. [19]. The following parameters were used for peak calling: -g mm -s 50 -p < 1E-5 -m 10 30 --broad -B –trackline:

-g: mappable genome size, defined as the genome size that can be sequenced. GRCm38/mm10 = 1.87E9 (G)

-s: size of sequencing tags

-p: *p*-value cut-off: 1E-5

-m: minimum length of called peak (10) and maximum gap allowed between two peaks (30) to be merged.

14: smallest peak size

—: input file format in BED format

Based on λlocal, MACS workflow uses dynamic Poisson distribution to calculate the *p*-value of the specific region based on the unique mapped reads. The region is defined as a peak when the *p*-value < 1E-5 (by default). The MACS-predicted peaks are also assigned enrichment scores. The more enriched these regions are, the more likely they represent true binding sites. In our analysis, MACS-predicted peaks were further filtered to obtain a list of true peaks (true *Ppp1r1b-lncRNA*-binding sites) at stringent enrichment score values of ≥10.

4. Peak mapping to promoter elements: To identify peaks that overlap with promoters, 25,111 coding and 3077 non-coding promoters for GRCm38/mm10 were downloaded from the Eukaryotic Promoter Database new (EPDnew) (https://epd.epfl.ch/epdnew/documents/MmNC_epdnew_001_pipeline.php, 1 March 2023) [20,21]. The EPDnew promoters are experimentally validated with next-generation sequencing-based whole-genome TSS mapping protocols, such as Cap Analysis of Gene Expression (CAGE) and Oligocapping, and include TATA-box, initiator motif (IM), CCAT-box, and other well-established promoter elements. Using the Bedtools “*intersect*” feature, true peaks (enrichment score ≥ 10) with at least 30% overlap with EPDnew promoters were identified.

5. Peaks mapping to putative enhancer elements: Putative enhancer elements were obtained from Enhancer Atlas Browser (Enhancer Atlas 2.0; http://www.enhanceratlas.org/indexv2.php) [22]. The database provides enhancer annotation in nine species, including human, mouse, fly, worm, zebrafish, rat, yeast, chicken, and boar annotations. The consensus enhancers were predicted based on multiple high-throughput experimental datasets (e.g., histone modification, CAGE, GRO-seq, transcription factor binding, and DHS). Currently, the updated database contains 6,198,364 enhancers and 7,437,255 enhancer-gene interactions involving 31,375 genes for 241 murine tissue/cell types identified from 5838 datasets such as NCBI GEO datasets, ENCODE project portal at UCSC, Epigenome Roadmap and FANTOM. As mentioned above, we used the Bedtools “intersect*”* feature to identify true peaks that overlapped with the enhancer elements at confidence score ≥ 1.

6. Peak visualization using UCSC Genome Browser and Broad Institute Integrative Genomics Viewer (IGV): The UCSC Genome Browser, which contains genome references assemblies for multiple species, was used to visualize and download *Ppp1r1b-lncRNA*-ChIRP derived peaks as well as specific genes and regions genome-wide. After selecting the GRCm38/mm10 genome on the UCSC genome browser, *Ppp1r1b-lncRNA*-ChIRP, control, and Peak Bed files were uploaded to custom tracks. The distribution of peaks across the genome and within specific regions was shown. IGV was used in a similar fashion for visualization and analysis [23].

7. Motif analysis using MEME-SEA: Motifs analysis was performed using Multiple EM for Motif Elicitation (MEME)-Simple Enrichment Analysis (SEA) v5.5.3. [24,25], using the following command line: sea —verbosity 5 —oc —thresh —align center —p *input_file* —m *motif_database* 10.0. MEME works by searching for repeated, ungapped sequence patterns that occur in the DNA or protein sequences provided by the user. The discovered motifs can be compared with databases of known motifs to identify matches to the motifs and display the motifs in various formats. The motif database used in this study is the Universal PBM Resource for Oligonucleotide Binding Evaluation (UniPROBE) database for the murine species, which is generated by universal protein binding microarray (PBM) assays on the in vitro DNA binding specificities of proteins [26].

#### 2.2.3. Functional Enrichment of Peaks’ Related Genes

1. Identifying peaks’ related genes: Data were downloaded from the UCSC Genome browser. To identify peaks’ related genes, we applied the following criteria: A. The reads are uniquely mapped to a protein-coding gene. B. The genes must be annotated (gene name present). C. The gene status is known.

2. Gene ontology annotation of peaks’ related genes: Gene Ontology (GO) analysis was used to predict the main biological functions that are enriched in the peaks’ related genes and assign them to specific molecular functions, biological processes, and cellular components [27]. All peaks’ related genes were mapped to GO terms in the database (http://www.geneontology.org). The number of genes for every term was then calculated. Finally, a hypergeometric test was used to find significantly enriched GO terms in the query list of peaks’ related genes. The calculated *p*-value goes through Bonferroni correction, and a corrected *p*-value ≤ 0.05 defines the significantly enriched GO terms in the peaks’ related genes.

3. KEGG pathway enrichment: To further understand the biological functions of the peak-related genes in a pathway-based contest, KEGG (Kyoto Encyclopedia of Genes and Genomes) was used [28]. This analysis identifies significantly enriched metabolic pathways or signal transduction pathways in peak-related genes compared with the target regions’ background. The analysis process follows the same pipeline as that in GO analysis.

### 2.3. Statistical Analysis

Quantified results and statistical parameters for each bioinformatic analysis step were presented with their data within the corresponding sections of the text.

## 3. Results

### 3.1. Quality Control and Alignment Statistics Results

**Figure 2:**
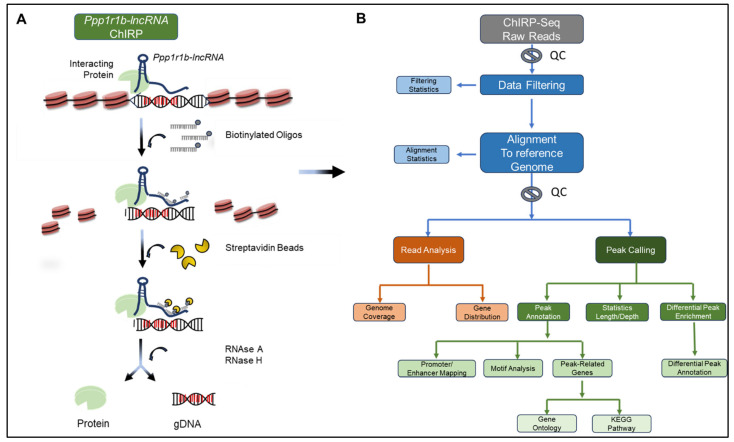
Schematic Summary of *Ppp1r1b-lncRNA*-ChIRP Assay and Bioinformatic Pipeline. (**A**) Chromatin was cross-linked *in vivo.* Biotinylated tiling probes were hybridized to target lncRNA, and chromatin complexes were purified using magnetic streptavidin beads, followed by stringent washes. The lncRNA-bound DNA or proteins were eluted with a cocktail of RNase A and H. A putative lncRNA binding DNA sequence is highlighted in red. Adopted from Chu et al. 2011. (**B**) The ChIRP-captured DNA fragments and control input libraries were subjected to next-generation sequencing. After quality control (QC) and alignment to the reference genome, the clean, uniquely mapped reads were used for peak calling and downstream bioinformatic analysis.

In this study, we mapped *Ppp1r1b-lncRNA* chromatin occupancy genome-wide by ChIRP-seq in a mouse myoblast cell line, which expresses endogenous *Ppp1r1b-lncRNA* [3,13]. Sequencing libraries were prepared from *Ppp1r1b-lncRNA*-ChIRP and control input DNA fragments and subjected to single read high throughput sequencing at a read length of 50 bp (Figure 2). An average of 38 million raw sequencing reads per sample were generated. After filtering low-quality reads and removing adaptor sequences, 36,534,935 and 38,429,369 (99.13% and 98.04%) clean reads were obtained from *Ppp1r1b-lncRNA*-ChIRP and control samples, respectively, to be used for downstream analysis. The sequencing data summary for each sample is summarized in Table 1.

For read mapping, the clean reads were mapped to the mouse reference genome GRCm38/mm10 using SOAPaligner/soap2 [18]. Only the alignments within 2 bp mismatches were considered.Strict quality control measures for each sample were applied, achieving a genome mapping rate of 95.21% and 96.97% for *Ppp1r1b-lncRNA*-ChIRP and control samples, respectively (Supplementary Materials, Table S1).

For peak calling, the uniquely mapped clean reads that only map to one genomic position in the total reads were included. Following this criteria, 31,664,010 and 32,579,793 uniquely mapped reads (86.7% and 84.78%) were obtained from *Ppp1r1b-lncRNA*ChIRP and control samples, respectively, to be used for downstream peak calling and subsequent analysis. Alignment statistics results and genome mapping rate for each sample are summarized in Table 2.

### 3.2. Genome and Gene Depth Distribution Analysis

**Figure 3:**
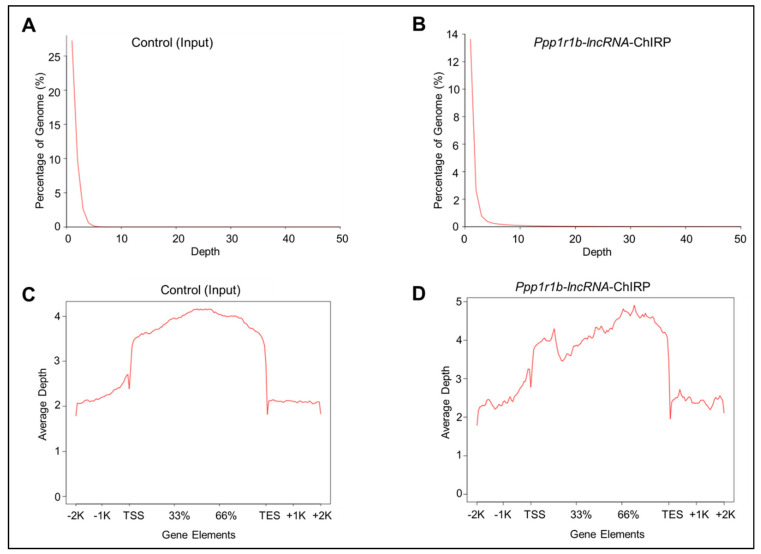
Genome and Gene Depth Distribution for Each Sample. (**A**,**B**) Composite genome coverage of *Ppp1r1b-lncRNA*-ChIRP and Control (input) sequencing reads in mouse myoblasts cell line. These figures represent all reads in each sample. (**C**,**D**) Composite sequencing reads profiles of *Ppp1r1b-lncRNA*-ChIRP and Control (input) samples across gene regions in mouse myoblast cell line. These figures only represent the reads mapped to gene elements. The ‘Input’ library was used as a control.

The uniquely mapped reads that passed quality control measures were then used to estimate the genome depth distribution for each sample separately using BEDTools. The percentage of genome coverage for each sample is shown in [Figure 3A,B]. Gene depth distribution was also obtained separately for each sample by BEDTools, and only those uniquely mapped reads were used in this analysis. As shown in [Figure 3C,D], the average depth of *Ppp1r1b-lncRNA*-ChIRP reads exhibited differential distribution in relation to genic regions with increased coverage around TSS and towards the distal 50% part of genes.

### 3.3. Peak Calling

The uniquely mapped reads were then used for genome-wide peak calling using MACS standard pipeline [19]. MACS-detected peak statistics at a *p*-value cut-off < 1E-5 are summarized in (Table 3). MACS peak calling statistics results include peak location, peak enrichment score, and peak length.

**Figure 4:**
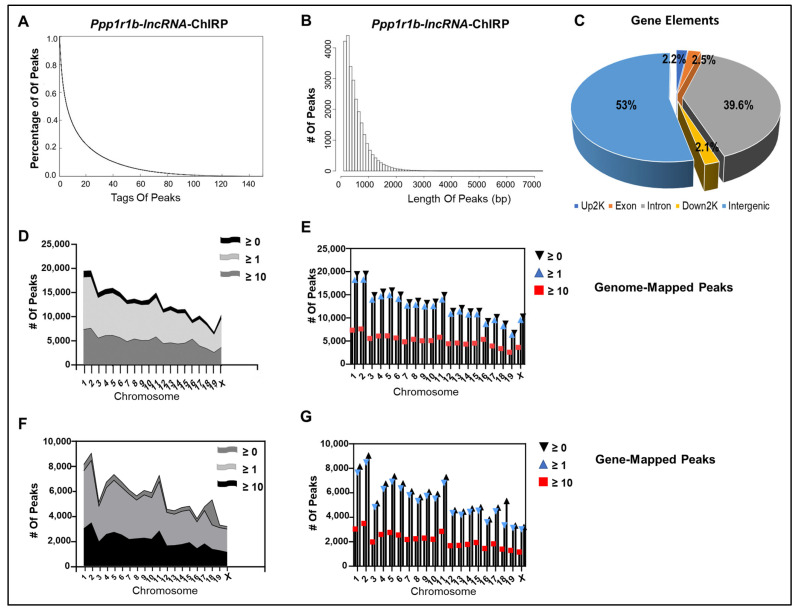
MACS-derived *Ppp1r1b-lncRNA*-ChIRP Peaks Statistics. (**A**) Depth Distribution of all peaks called by MACS at *p*-value < 1E-5. The *X*-axis indicates the number of reads, and the *Y*-axis indicates the proportion of peaks in the specific number of reads. (**B**) Length distribution of all MACS-called peaks. The *X*-axis refers to peak length, and the *Y*-axis refers to peak numbers. (**C**) Distribution of all MACS-called peaks based on genomic position and gene elements: intergenic, introns, downstream, upstream, and exons. (**D**,**E**) Number of MACS-called Peaks (*p* < 1E-5) genome-wide (**D**) and on each chromosome (**E**) at different enrichment score cut-off values (≥0, ≥1, and ≥10). The *X*-axis indicates chromosome number, and the *Y*-axis indicates the number of peaks mapped to each chromosome. (**F**,**G**) Number of MACS-called peaks (*p* < 1E-5) that mapped to known protein-coding genes on all chromosomes (**F**) and on each chromosome (**G**) at different enrichment score cut-off values (≥0, ≥1, and ≥10). The *X*-axis indicates chromosome number, and the *Y*-axis indicates the number of gene-mapped peaks on each chromosome.

In total, MACS identified 261,455 peaks in the *Ppp1r1b-lncRNA*-ChIRP sample that passed a *p*-value < 1E-5 against control input. *Ppp1r1b-lncRNA-ChIRP* peaks were short and focal, ranging between 165 and 4500 bp in length with a mode of 165 bp and an average peak length of 558 bp (Figure 4A,B). With a genome coverage rate of 5.35%, the peaks were widely distributed in the intergenic (53.4% of all peaks) and the genic (46.4% of all peaks) regions. With reference to the gene elements, 39.6% of the gene-mapped peaks were located in introns, while 2.5% were mapped to exons, 2.2% mapped to immediate Up2k, and 2.1% mapped to immediate Down2K, of the TSS and TES, respectively (Figure 4C).

**Figure 5:**
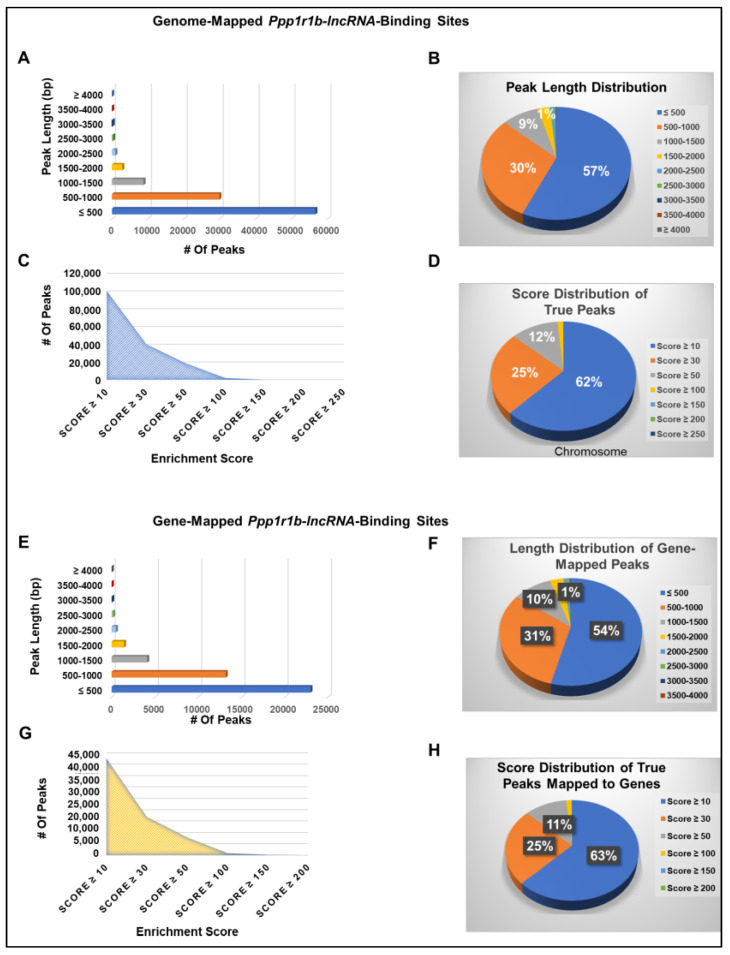
*Ppp1r1b-lncRNA*-Binding Sites (True Peaks) Statistics. (**A**) Number of genome-mapped true peaks at different peak length (bp) values. (**B**) Distribution of genome-mapped true peaks at different peak length (bp) values. (**C**) Number of genome-mapped true peaks at different enrichment score values. (**D**) Distribution of genome-mapped true peaks at different enrichment score values. (**E**) Number of gene-mapped true peaks at different peak length (bp) values. (**F**) Distribution of gene-mapped true peaks at different peak length (bp) values. (**G**) Number of gene-mapped true peaks at different enrichment score values. (**H**) Distribution of gene-mapped true peaks at different enrichment score values.

To enhance the specificity of peaks that represent true *Ppp1r1b-lncRNA* binding sites, only peaks with enrichment scores equal to or greater than 10 were defined as true peaks that represent *Ppp1r1b-lncRNA*-binding sites and retained for further analysis. Using these parameters, 99,732 true *Ppp1r1b-lncRNA*-binding sites were identified genome-wide, of which 42,393 (43% of true peaks) were mapped to protein-coding genes, indicating that the ratio of the gene-mapped peaks to the total number of peaks did not change significantly despite using more stringent thresholds for true peaks selection. Notably, like the genome-mapped binding sites (Figure 4D,E), the gene-mapped binding sites (Figure 4F,G) were widely spread on all chromosomes and retained similar patterns of distribution at different cut-off values for enrichment scores. Furthermore, the length distributions, as well as enrichment score proportions of the genome-mapped binding sites (Figure 5A–D) were comparable to those mapped to the genes (Figure 5E–H). The peak calling results were stored in wiggle files and viewed on the UCSC genome browser and the Integrative Genome Viewer (IGV) for peak visualization. Examples of *Ppp1r1b-lncRNA*-binding sites are presented in (Supplementary Materials, Figure S1).

### 3.4. Functional Annotation of Peaks’ Related Genes

**Figure 6:**
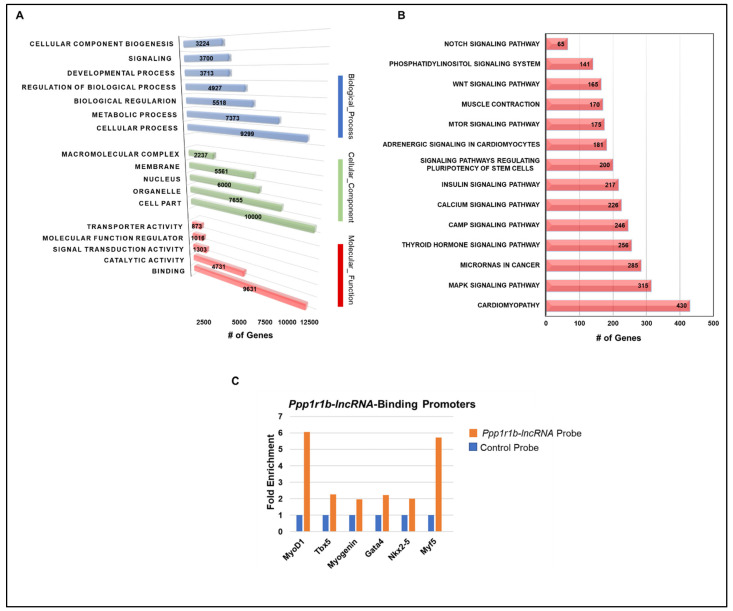
Functional Annotation of *Ppp1r1b-lncRNA*-Binding Sites. (**A**) Gene Ontology (GO) analysis of *Ppp1r1b-lncRNA*-ChIRP peaks’ related genes. The top significantly enriched GO terms (FDR < 0.05) involved in biological processes, cellular components, or molecular functions are presented. The number of genes in each term is shown. (**B**) Summary of KEGG Pathway analysis of *Ppp1r1b-lncRNA*-ChIRP peaks’ related genes. The top significantly enriched pathways are presented. The number of genes in each pathway is shown. Only true peaks’ related genes are included in these analyses. (**C**) *Ppp1r1b-lncRNA*-ChIRP-PCR validation of *Ppp1r1b-lncRNA* interaction with promoters of myogenic differentiation transcription factors. A LacZ probe was used as a negative control.

The peaks’ related genes are candidate *Ppp1r1b-lncRNA* binding sites, from which we may infer its potential biological impact and mechanisms of function. The true peaks’ related genes, which define *Ppp1r1b-lncRNA*-binding sites, were listed for functional annotation to characterize the functional properties of these genes and their products using GO analysis. At a global level, *Ppp1r1b-lncRNA* binding sites were enriched in molecular function in terms of binding, catalytic activity, transcription regulation, and signal transduction. The nucleus, cellular organelles, and cellular membrane were enriched cellular components. Biological regulation, cellular biogenesis, metabolic process, and cellular transport were enriched biological processes (Figure 6A).

To elucidate *Ppp1r1b-lncRNA* interactions within certain biological contexts or signaling pathways, KEGG pathway analysis was also performed on all *Ppp1r1b-ncRNA* binding sites’ related genes (Figure 6B, Table 4). Pathways of cardiomyopathy, cancer and pluripotency, transcriptional regulation, and developmental pathways were among the top enriched processes. Further, critical signaling pathways of development and myogenesis, including Wnt, Notch, metabolism, and insulin signaling, were among the top enriched signaling pathways.

### 3.5. Promoter Mapped Peaks

As previously demonstrated [13], *Ppp1r1b-lncRNA* executes its functions through the interaction with promoters of myogenic transcription factors. Further, *Ppp1r1b-lncRNA*-ChIRP peaks (binding sites) are mostly narrow, reminiscent of TF binding sites. Based on these observations, we performed independent ChIRP-PCR assays. In addition to the previously known interactions with *MyoD1* and *Tbx5*, we validated four new interactions with myogenic differentiation factors specific to cardiac and skeletal myocytes identified from ChIRP-seq (Figure 6C). We further examined *Ppp1r1b-lncRNA* binding to promoter regions using EPDnew [20,21], including all peaks in this analysis. In total, 2871 peaks were mapped to experimentally annotated promoter sequences. Of these, 1180 true peaks (enrichment score ≥ 10) were retained as promoter-mapped *Ppp1r1b-lncRNA*-binding sites, accounting for 28% of the true binding sites that mapped to protein-coding genes.

Notably, the promoter-mapped binding sites were significantly enriched with transcription regulators, including those involved in Wnt signaling pathway (*Lef1* and *Tcf7*), heart muscle development (*Gata4* and *Mef2c*) regulation of transcription by RNA Pol-lI (*Sox17*, *SRF*, *EGR2*) and chromatin modification (*Smarcb1* and *Taf9*). Correspondingly, the binding sites that mapped to these genes had high enrichment scores (Table 5). Intriguingly, up to 80% of the identified promoter-mapped binding sites were enriched with one or more of the previously validated sequence elements of proximal promotes (TATA-box, transcription initiator motif, CCAT-box, and GC-box) with established specificity for transcription initiation by RNA Pol-II (Table 5). Together, these results are consistent with *Ppp1r1b-lncRNA* function in transcription initiation of myogenic regulators via binding to their promoter elements.

**Figure 7:**
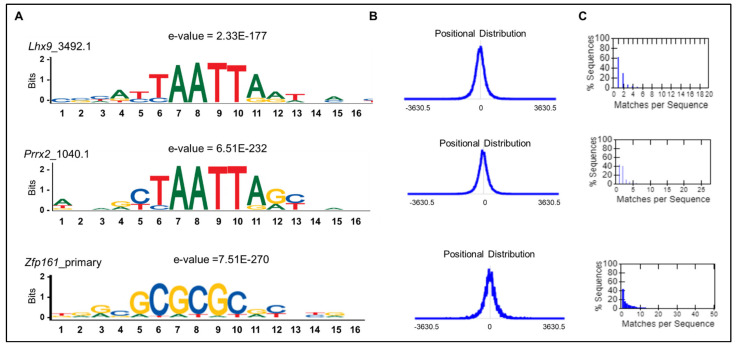
Motif Enrichment in *Ppp1r1b-lncRNA* Binding Sites to Transcription Factors. (**A**) Representative examples of significantly enriched motifs in *Ppp1r1b-lncRNA*-binding sites in Homeobox transcription factors (*Lhx9* and *Prrx2* with TA-rich motifs) and zinc fingers (*Zfp161* and *Zbtb14* with GC-rich motif). (**B**) Positional distribution of the best match to the motif in the primary sequences. The plot is smoothed with a triangular function whose width is 5% of the maximum primary sequence length. The position of the dotted vertical line indicates whether the sequences were aligned on their left ends, centers, or right ends, respectively. (**C**) The percentage of sequences matching the motif. A sequence is said to match the motif if some position within it has a match score greater than or equal to the optimal score threshold.

LncRNA binding of transcription factors is mainly governed by their sequence specificity and, therefore, is typically associated with highly localized ChIRP-Seq signals in the genome. Therefore, we furthered our motif analysis using MEME: Simple Enrichment Analysis, including all *Ppp1r1b-lncRNA*-binding sites. Using this analysis, we identified 310 transcription factors with specific motif sequences. Of these transcription factors, 25% belong to the Homeobox family, such as *HOX* and *LHX*, and were enriched with TA-rich motifs, such as TTAATTAAT and TAATTA motifs (Figure 7, Table 6). In addition, a few zinc finger-related transcription factors were enriched with GC sequence repeats (Figure 7, Table 6). Together, these findings suggest novel motif sequences for *Ppp1r1b-lncRNA*-specific interactions with transcription factors.

### 3.6. Enhancer Mapped Peaks

Cell and tissue specificity are governed by tissue/cell-specific enhancer elements. Using Enhancer Atlas 2.0 workflow [22], all MACS-derived peaks were mapped to detect the enhancer elements that may be enriched in *Ppp1r1b-lncRNA*-ChIRP signals and to identify their tissue/cell-specific enrichment. In total, more than 12,000,000 enhancer sequences were mapped to all peaks. By applying stringent filtering, only enhancers that are enriched in the true *Ppp1r1b-lncRNA*-binding sites at confidence score ≥ 1 and ≥30% overlap with a given enhancer were retained, leading to 136,521 *Ppp1r1b-lncRNA*-bound enhancer consensuses. Among these signals, 12% (16571 enhancers) showed specific enrichment in cardiac progenitor cells, fetal heart, and limb tissues at high confidence scores (Supplementary Materials, Table S2). These findings correspond to *Ppp1r1b-lncRNA*-specific cellular function in myogenic differentiation of heart and muscle development. Furthermore, histone structure genes and epigenetic modification process were enriched in the enhancers’ enriched *Ppp1r1b-lncRNA*-binding genes.

## 4. Discussion

In this study, we applied ChIRP-seq technology against GRCm38/mm10 murine species to identify *Ppp1r1b-lncRNA* chromatin occupancy genome-wide in mouse myoblast cell line, which expresses *Ppp1r1b-lncRNA* [3,13]. As described previously [16,17], using Glutaraldehyde crosslinking and *Ppp1r1b-lncRNA*-targeted high-affinity probe, the lncRNA-bound DNA sequences were recovered and purified. LacZ probe was used as a negative control, and no cross-hybridization with *Ppp1r1b-lncRNA* was observed. The purified *Ppp1r1b-lncRNA*-ChIRP DNA fragments were used to generate the sequencing libraries and subjected to high throughput single-read sequencing. An input DNA sample was subjected to the same sequencing protocol and used as a control to allow interpretation of the results.

We selected MACS, a window-based method [19], for peak calling based on previous knowledge that *Ppp1r1b-lncRNA* executes its function via the interaction with myogenic transcription factors [13]. MACS has been reported to outperform several other methods in the identification of transcription-binding sites that tend to be focal and narrow [16,19]. In addition, the MACS pipeline is user-friendly and provides important information for each peak, including genomic position, enrichment score, etc.

Using MACS, we identified 244,944 *Ppp1r1b-lncRNA*-ChIRP peaks in the genome at *p* < 1E-5 and enrichment score ≥ 1. *Ppp1r1b-lncRNA*-ChIRP peaks were focal and narrow, averaging 554 bp in length, and typically span less than 300 hundred nucleotides (Mode 165 bp) but occasionally stretched beyond 2K BPs (1% of all peaks). We found the peaks mapped to the intergenic regions (53.6%) and gene regions (47.4%) and distributed on all chromosomes (Figure 4C). By applying a more stringent enrichment threshold (score ≥ 10) for true peaks, a total of 99,732 *Ppp1r1b-lncRNA* binding sites were detected at high confidence, of which 44% mapped to annotated protein-coding genes. Hence, despite applying stringent criteria to define true peaks, the proportion of binding sites that mapped to the protein-coding genes was retained, and the distribution patterns of the peaks on the different chromosomes remained consistent at different cut-off values for peak length and enrichment scores, both at genes and genome scales.

The enrichments with myogenesis, muscle contractions, and cardiomyopathy in the interacted genes reinforce the essential role of *Ppp1r1b-lncRNA* in myogenic differentiation. Other than myogenic differentiation factors, Wnt signaling, Notch signaling, and multipotency pathways are also critical to the lineage commitment of skeletal muscle and cardiac progenitors. Moreover, we identified enrichment with sarcomere structures genes (*Myh7*, *Tnnt2*, and *Tcap*) (Table 4) and components of myocyte membrane, including the Dystrophin–Glycoprotein complex (DGC) components (*Dmd, Dnta, Dntb*, Sgcd, and *Utrn*) that play important roles in maintaining the integrity of myocyte cellular membranes in heart and skeletal muscles (Supplementary Materials, Table S3).

The enrichment with ribonucleoproteins and RNA binding proteins (*Hnrnpa1*, *Rbm20*, *Rbfox1*) known to be involved in cardiac and muscle diseases and with chromatin modification genes (*Kdm3b*, *Kdm5c*, and *Hdac4*) supports that *Ppp1r1b-lncRNA* roles may span transcriptional/post-transcriptional regulation and chromatin modifications (Supplementary Materials, Table S3). These newly identified candidates at the genomic scale beget further functional studies. In addition, we observed numerous specific *Ppp1r1b-lncRNA*-binding sites that mapped to other annotated regulatory non-coding RNA (*St7*) and micro-RNA (*Mir466* and *Mir1191*) genes of known functions, with signal intensities and enrichment scores comparable to those mapped to the protein-coding genes (Supplementary Materials, Table S3). Thus, the current comprehensive repertoire of *Ppp1r1b-lncRNA* occupancy provides a rich resource for a complete understanding of *Ppp1r1b-lncRNA* function.

Importantly, among the *Ppp1r1b-lncRNA-binding* sites, we identified the previously confirmed *Ppp1r1b-lncRNA*-interactions with *TBX5* and *MyoD1* using ChIRP-PCR [13] (Figure 6C), supporting that our criteria for detecting *Ppp1r1b-lncRNA*-binding sites can identify true signals with potential functional relevance. Intriguingly, we also detected new unique interactions with other key transcription factors of myogenic differentiation in heart and skeletal muscles and validated these new findings independently using ChIRP-PCR as a gold standard (Figure 6C).

By mapping the *Ppp1r1b-lncRNA* binding sites to the experimentally validated promoters of the EPDnew database, we identified 1180 hits located in experimentally validated promoters within −1000 to +/−200 kb of TSS of a given gene. These signals predict true promoter-mapped *Ppp1r1b-lncRNA*-binding sites based on enrichment score ≥ 10. Importantly, most of these promoter occupancy sites were enriched with one or more of the four previously annotated regulatory elements that define proximal promoters with binding affinity to RNA Pol-II. This pattern of *Ppp1r1b-lncRNA* interaction with the promoter’s elements supports the idea that *Ppp1r1b-lncRNA* may promote transcriptional initiation [29,30] (Table 5). These data also demonstrate that ChIRP-seq may precisely uncover biologically relevant interactions.

As stated previously, the observed *Ppp1r1b-lncRNA*-ChIRP peak pattern is similar to ChIP-seq peaks of transcription factors binding sites. It also resembles the pattern of HOTAIR-ChIRP, a lncRNA known to recruit PRC2 [16]. Like transcription factors, it has been postulated that specific DNA motifs may serve to facilitate lncRNA selective interactions, introducing a new class of regulatory elements in the genome that are specifically targeted by lncRNA. For instance, a GA-rich homopurine motif was previously reported for HOTAIR binding [16]. However, unlike HOTAIR, *Ppp1r1b-lncRNA* has been shown to interfere with PRC2 binding at the promoter of myogenic transcription factors [13]. Collectively, these different interaction motifs may indicate that *Ppp1r1b-lncRNA* binding and the resulting function involve a coordinated action of multiple factors. Therefore, identifying motifs that infer specificity for *Ppp1r1b-lncRNA* interactions with chromatin may lead, at a mechanistic level, to classify lncRNAs that alter chromatin states in a specific manner (recruiting PRC2 to promoter vs. inhibiting PRC2 binding at promoter). Indeed, using MEME-SEA, we identified TA-rich motifs occupying *Ppp1r1b-lncRNA*-binding sites in the Homeobox family of transcription factors. This finding may suggest other functions for *Ppp1r1b-lncRNA* and raises particular interest in future studies since the Homeobox proteins play critical roles in organogenesis and patterning, including cardiogenesis, during development.

Although *Ppp1r1b-lncRNA*-ChIRP peaks were narrow and focal, they were not restricted to proximal promoters, but a large proportion of them were located within the intronic and intergenic areas, suggesting potential enrichment with other regulatory elements, such as enhancers. As distal cis-regulatory elements, enhancers activate the transcription of their target genes in cell type-specific and tissue-specific manners [31,32]. To date, the Enhancer Atlas 2.0 database [23] is the most comprehensive enhancer database that includes 13,494,603 annotated consensus enhancers based on 16,055 datasets in 586 tissue/cell types across nine species. Indeed, we identified significant enrichment with sequence motifs of distal enhancers, with more than 90% possessing cell type/tissue-specificity. By narrowing our analysis to cardiac and limb cell type/tissue, we identified 3390 enhancers at a confidence score >1 and overlapped with *Ppp1r1b-lncRNA*-binding sites ≥ 30%.

## 5. Conclusions

Our study provides *Ppp1r1b-lncRNA* occupancy at a genome scale. The identified interaction with promoters and enhancers and their putative enriched motifs may potentially dictate *Ppp1r1b-lncRNA* function in myogenic differentiation and potentially other cellular and biological processes. We should acknowledge the limitations of our study. Despite the comprehensive analysis and the new insights, our study remains descriptive, and the biological impacts of the newly identified interactions in altering chromatin state and influencing target gene expression remain to be mechanistically investigated. Pending functional results of selected important candidates, the study will further our understanding of the *Ppp1r1b-lncRNA*-derived functional regulome that may dictate its essential role in myogenic differentiation and potentially other cellular and biological processes.

## Figures and Tables

**Table 1:** Summary of sequencing data for each sample.

Sample ID	Fragment Length (bp)	Sequencing Strategy	Clean Reads Number	Clean Data Size (bp)	Clean Rate (%)
*Ppp1r1b-lncRNA*_ChIRP	100–500	SE50	36,534,935	1.83E+09	99.13
Control	100–500	SE50	38,429,369	1.92E+09	98.04

Fragment Length (bp): DNA fragment for library building; SE50: Strategy of sequencing sample with single end (SE), and the following number reflects read length. Clean Reads Number: The count of reads number in clean data. Clean Data Size: The count of bases in clean data. Clean Rate (%): The ratio of clean data size to raw data size = Clean Data Size (bp)/Raw Data Size (bp).

**Table 2:** Alignment statistics results and genome mapping rate for each sample.

Sample ID	Species	Clean Reads	Mapped Reads	Mapped Rate (%)	Uniquely Mapped Reads	Uniquely Mapped Rate (%)
*Ppp1r1b-lncRNA*-ChIRP	mm10	36,534,935	34,783,233	95.21	31,674,010	86.7
Control	mm10	38,429,369	37,264,694	96.97	32,579,793	84.78

Clean Reads: Total clean reads number; Mapped Reads: Total reads that can be mapped to the reference genome. Mapped Reads Rate (10%): The proportion of reads that can be mapped to the reference genome in total reads. Uniquely Mapped Reads: Total reads that only map to one position in the reference genome. Uniquely Mapped Rate (%): The proportion of reads that only map to one position in total reads.

**Table 3:** Peak calling statistics.

Sample ID	Peak Number	Total Length	Average Length	Total Tag Depth	Average Tag Depth	Genome Rate (%)
*Ppp1r1b-lncRNA*-ChIRP	261,455	146,128,139	558	3,848,968.705	14	5.35

Peak number: Number of all MACS-detected peaks. Total Length: Total length of all peaks. Average Length: Average length of all peaks. Total Tag Depth: Total tag depth of all summits. Average Tag Depth: Average tag depth of all summits. Genome Rate: Proportion of total length of all peaks in the whole genome.

**Table 4:** KEGG pathway analysis. Top 20 significant pathways enriched in the peaks’ related genes. *p*-values, adjusted *p*-values, and five representative genes in each category are presented.

KEGG Pathway	*p* Value	adj*p*-Value	Example Genes
Cardiomyopathy	9.74E-10	5.29E-08	*Myh6*, *Tnnt2*, *Tnni3*, *Tcap*, *DMD*
MAPK Signaling Pathway	7.83E-09	2.55E-07	MAPK12, MAPK10, MAPK1, BMP4, Ppp2cb
MicroRNAs in cancer	6.26E-08	1.46E-06	Ezr, Tnr, Sos1, Lrp1, Abl1
Thyroid hormone signaling pathway	6.26E-08	1.46E-06	*Adam23*, *Prkca*, *Med12l*, *Otog*, *Bmp4*
CAMP signaling pathway	1.24E-07	2.70E-06	*Atp6v1h*, *Rdh10*, *Ndufs1*, *Ndufa10*, *Prim2*
Calcium signaling pathway	1.24E-07	2.70E-06	*Cacna1c*, *Tnnc2*, *Cacna1d*, *Plcb1*, *Pde1c*
Insulin resistance	1.83E-07	3.73E-06	*Prcke*, *nfkb1*, *Prkag2*, *Slc27a1*, *Ppara*
Regulating pluripotency of stem cells	3.19E-07	5.47E-06	*Meis1*, *Jak2*, *Lhx2*, *cdh1*, *Pou5f1*
Adrenergic signaling of cardiomyocytes	1.12E-05	1.21E-04	*Cacng3,Lam4*, *Tnn*, *Ctnna*, *Lamc1*
mTOR signaling pathway	2.36E-05	2.34E-04	*Grb10*, *Rheb*, *Mtor*, *Deptor*, *Akt1,3*
Muscle contraction	8.21E-05	6.08E-04	*Atp1a4*, *cacng2*, *Casq2*, *Ryr2*, *Cox7a21*
Wnt signaling pathway	8.54E-05	6.18E-04	*Lef1*, *Wnt3*, *Tcf7*, *Nfatc1*, *fzd6,3,9*
Inositol phosphate metabolism	8.54E-05	6.18E-04	*Pten*, *Mtmr2*, *Plcb1*, *Itpk1*, *Pip4k2b*
Notch signaling pathway	0.000121564	8.62E-04	*Notch3*, *Jag1*, *EP300*, *Hess*, *Kat2a*
Regulation of actin cytoskeleton	0.000134814	9.16E-04	*Vwc2l*, *Lamb3*, *Cpne4*, *Adam23*, *Actn1*

**Table 5:** Promoter-mapped *Ppp1r1b-lncRNA*-binding sites involved in transcription by RNA Pol-II. Top 25 experimentally validated *Ppp1r1b-lncRNA*-bound promoters involved in transaction by RNA Pol-II. Neg: Negative; Pos: Positive.

Peak Chr	Peak Start	Peak End	PG + Peak ID	Peak Score	Promoter Start	Promoter End	Gene Symbol	TATA	IM	CCAAT	GC	Strand	Overlap vs. Promoter	Overlap vs. Promoter (%)
chr14	63,244,293	63,245,511	77,537	74.66	63,245,227	63,245,287	Gata4		X	X	X	Neg	60	100
chr13	83,523,709	83,524,546	108,425	10.09	83,524,504	83,524,564	Mef2c	X	X	X		Pos	60	100
chr17	46,555,824	46,556,722	108,938	32.96	46,556,158	46,556,218	Srf	X		X	X	Neg	60	100
chr15	79,345,918	79,346,730	107,952	21.98	79,346,563	79,346,623	Maff		X	X		Pos	60	100
chr10	81427146	81,427,779	108,158	14.82	81,427,146	81,427,206	Nfic		X	X		Neg	60	100
chr1	135,257,953	135,258,882	12,555	36.78	135,258,446	135,258,506	Elf3	X	X	X		Neg	60	100
chr1	4,493,533	4,493,908	108,119	15.73	4,493,597	4,493,657	Sox17	X	X	X	X	Neg	60	100
chr10	67,536,485	67,538,050	25,893	55.61	67,537,820	67,537,880	Egr2	X				Pos	60	100
chr17	34,031,775	34,032,425	107,642	71.94	34,032,319	34,032,379	Rxrb				X	Pos	60	100
chr14	79,479,972	79,481,156	108,477	10.58	79,481,129	79,481,189	Elf1	X				Pos	27	45
chr12	56,534,915	56,535,714	107,998	20.16	56,535,187	56,535,247	Nkx2-1				X	Neg	60	100
chr7	19,629,319	19,629,812	211,713	38.52	19,629,412	19,629,472	Relb			X	X	Neg	60	100
chr15	102,625,143	102,625,696	94,380	99.04	102,625,475	102,625,535	Atf7				X	Neg	60	100
chr1	51,986,786	51,987,935	4273	28.28	51,987,077	51,987,137	Stat4		X	X	X	Pos	60	100
chr3	131,108,912	131,111,373	161,236	23.35	131,110,273	131,110,333	Lef1				X	Pos	60	100
chr2	28,621,840	28,622,035	132,357	24.47	28,621,934	28,621,994	Gfi1b			X		Neg	60	100
chr3	30,138,874	30,140,715	151,247	36.8	30,140,412	30,140,472	Mecom			X		Neg	60	100
chr16	10,992,402	10,993,184	108,188	13.97	10,993,068	10,993,128	Litaf		X	X	X	Neg	60	100
chr7	4,915,143	4,915,650	108,366	10.16	4,915,172	4,915,232	Zfp628				X	Pos	60	100
chr5	134,306,110	134,306,838	193,226	34.41	134,306,598	134,306,658	Gtf2i			X		Neg	60	100
chr13	100,651,310	100,651,684	108,247	12.71	100,651,575	100,651,635	Taf9		X	X		Pos	60	100
chr10	75,921,059	75,921,711	26,848	31.22	75,921,567	75,921,627	Smarcb1			X		Neg	60	100
chrX	12,761,915	12,762,355	251,942	39.97	12,762,005	12,762,065	Med14				X	Neg	60	100
chr7	139,943,548	139,943,869	107,961	21.67	139,943,772	139,943,832	Utf1				X	Pos	60	100
chr3	52,104,975	52,105,552	153,279	30.45	52,104,980	52,105,040	Maml3		X		X	Neg	60	100

**Table 6:** Motif enrichment of *Ppp1r1b-lncRNA*-bound transcription factors. Top 20 *Ppp1r1b-lncRNA*-bound transcription factors with enriched motif sequences.

RANK	ID	ALT_ID	CONSENSUS	SCORE_THR	PVALUE	EVALUE	QVALUE
79	UP00237_1	Otp_3496.1	VVYWRTTAATTAAYDNG	4.2	0.00E+00	7.69E-306	0.00E+00
87	UP00164_1	Hoxa7_2668.2	SGMNTTAATTAATDNNC	7.5	2.01E-273	7.76E-271	1.70E-273
128	UP00175_1	Lhx9_3492.1	CBYATTAATTAATHMCY	6.1	6.04E-180	2.33E-177	3.47E-180
69	UP00144_1	Hoxb4_2627.1	CNNRTTAATTAATWAHY	8.3	2.81e-343	1.08e-340	1.08e-340
7	UP00169_1	Lmx1b_3433.2	VDWWWTTAATTAATWHB	6.6	3.33e-1146	1.28e-1143	1.28e-1143
40	UP00078_1	Arid3a_primary	SNNHTTAATTAAAMNHN	7.8	3.38e-506	1.30e-503	1.30e-503
55	UP00141_1	Vsx1_1728.1	CSARTTAATTAAYNAHT	7.8	3.96e-399	1.53e-396	1.53e-396
78	UP00209_2	Cart1_1275.1	BVMNTTAATTAAYYNNN	6.7	1.83e-310	7.05E-308	1.72e-310
18	UP00129_1	Pou3f1_3819.1	DVNTAATTAATTAABTN	6.7	6.07e-920	2.34e-917	2.34e-917
70	UP00142_1	Uncx4.1_2281.2	VNTAATTAATTAABGSG	7.3	6.51e-337	2.51e-334	2.51e-334
51	UP00172_1	Prop1_3949.1	VGVRTTAATTAAKWNNC	7.3	6.71e-422	2.59e-419	2.59e-419
47	UP00196_1	Hoxa4_3426.1	DDTTATTAATTAACKBG	6.2	7.06e-449	2.72e-446	2.72e-446
81	UP00182_1	Hoxa6_1040.1	AMGKTAATTACCHHAD	9.1	7.54E-304	2.91E-301	6.86E-304
82	UP00189_1	Hoxa5_3415.1	AMGKTAATTAVCWHAD	7.5	2.19E-300	8.45E-298	1.97E-300
85	UP00248_1	Pax7_3783.1	MSHNYTAATTARBHVDN	10	3.56E-284	1.37E-281	3.08E-284
92	UP00174_1	Hoxa2_3079.1	AVGGTAATTASCHMAN	7.4	9.71E-260	3.75E-257	7.77E-260
94	UP00214_1	Hoxb5_3122.2	ANGKTAATTASCHMAT	9.1	2.45E-257	9.45E-255	1.92E-257
97	UP00167_1	En1_3123.2	RNNAACTAATTARKDC	5.8	3.29E-249	1.27E-246	2.50E-249
88	UP00065_1	Zfp161_primary	KGGCGCGCGCRCHYRD	14	1.94E-272	7.51E-270	1.63E-272
193	UP00001_1	E2F2_primary	NHWARGGCGCGCSAH	21	1.65E-74	6.38E-72	6.31E-75

## Data Availability

The datasets used and analyzed during the current study are available from the corresponding author upon reasonable request. The study was registered in the Gene Expression Omnibus (GEO) repository [www.ncbi.nlm.nih.gov/geo] under [Neonatal Heart Maturation (NHM) SupperSeries GSE85728. http://www.ncbi.nlm.nih.gov/geo/query/acc.cgi?acc=GSE85728], accessed on 20 August 2016. Upon acceptance of this manuscript, ChIRP sequencing data will be deposited to the repository and made publicly available. C2C12 Cell Line Source: In this study we employed a mouse myoblast cell line obtained from ATCC. https://www.atcc.org/products/crl-1772 (CRL-1772 ™). This line is well-established, commercially available, and has been used by researchers for more than a decade [13].

## Supplementary Materials

The following supporting information can be downloaded at: https://www.mdpi.com/article/10.3390/cells12242805/s1, Figure S1: *Ppp1r1b-lncRNA* Binding Sites to Transcription Factors. A. IGV viewer windows depict *Ppp1r1b-lncRNA*-sites (peaks) enriched in the promoter, intronic, and the distal 50% regions of genes, including myogenic differentiation transcription factors, Homeobox transcription factors (TA-rich motifs) and zinc fingers (GC-rich motif). Table S1: QC results for each sample; Table S2: *Ppp1r1b-lncRNA*-bound enhancers. Top 100 *Ppp1r1b-lncRNA*-enriched enhancers with specificity to fetal heart- or muscle- tissue/cells; Table S3: Examples of Genome-wide *Ppp1r1b-lncRNA* Interactions. Examples of the Dystrophin-Glycoprotein Complex (DGC) genes, Chromatin Modification genes, RNA-binding protein genes, and noncoding RNA genes are presented.
