# Supplementary material for: Mapping Chromatin Occupancy of Ppp1r1b-lncRNA Genome-Wide Using Chromatin Isolation by RNA Purification (ChIRP)-seq
Source: Cells. 2023 Dec 8;12(24):2805. doi: 10.3390/cells12242805 (PMC10741483; doi:10.3390/cells12242805)
Supplement: Supplementary file 1 [file cells-12-02805-s001.zip › cells-2708232-supplementary.pdf]

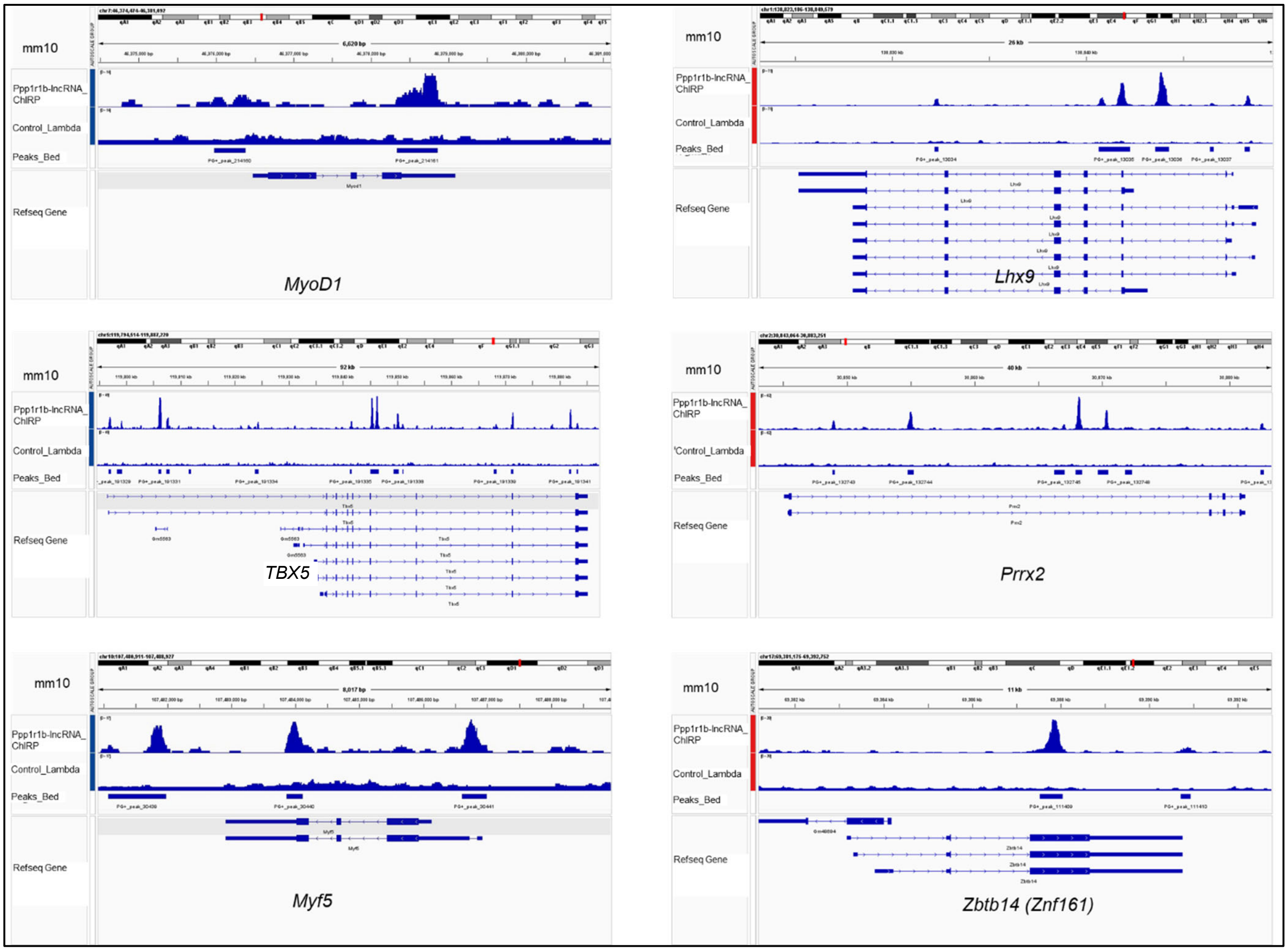

Supplementary Figure S1. *Ppp1r1b-lncRNA* Binding Sites to Transcription Factors. A. IGV viewer windows depict *Ppp1r1b-lncRNA*-sites (peaks) enriched in the promoter, intronic, and the distal 50% regions of genes, including myogenic differentiation transcription factors, Homeobox transcription factors (TA-rich motifs) and zinc fingers (GC-rich motif).
